# On the evolution of omnivory in a community context

**DOI:** 10.1002/ece3.923

**Published:** 2013-12-29

**Authors:** Alex M Chubaty, Brian O Ma, Robert W Stein, David R Gillespie, Lee M Henry, Conan Phelan, Eirikur Palsson, Franz W Simon, Bernard D Roitberg

**Affiliations:** 1 Department of Biological Sciences, Simon Fraser University8888 University Drive, Burnaby, British Columbia, V5A1S6, Canada; 2 ESSA Technologies Ltd.Vancouver, British Columbia, V6H 3H4, Canada; 3 Agriculture and Agri-Food Canada6947 Highway 7, PO Box 1000, Agassiz, British Columbia, V0M 1A0, Canada; 4 Department of Zoology, University of OxfordSouth Parks Road, Oxford, OX1 3PS, UK

**Keywords:** Community structure, density-dependent, diet choice, evolutionary game, frequency-dependent, genotype–environment interaction, omnivory, optimal foraging, phenotype

## Abstract

Omnivory is extremely common in animals, yet theory predicts that when given a choice of resources specialization should be favored over being generalist. The evolution of a feeding phenotype involves complex interactions with many factors other than resource choice alone, including environmental heterogeneity, resource quality, availability, and interactions with other organisms. We applied an evolutionary simulation model to examine how ecological conditions shape evolution of feeding phenotypes (e.g., omnivory), by varying the quality and availability (absolute and relative) of plant and animal (prey) resources. Resulting feeding phenotypes were defined by the relative contribution of plants and prey to diets of individuals. We characterized organisms using seven traits that were allowed to evolve freely in different simulated environments, and we asked which traits are important for different feeding phenotypes to evolve among interacting organisms. Carnivores, herbivores, and omnivores all coexisted without any requirement in the model for a synergistic effect of eating plant and animal prey. Omnivores were most prevalent when ratio of plants and animal prey was low, and to a lesser degree, when habitat productivity was high. A key result of the model is that omnivores evolved through many different combinations of trait values and environmental contexts. Specific combinations of traits tended to form emergent trait complexes, and under certain environmental conditions, are expressed as omnivorous feeding phenotypes. The results indicate that relative availabilities of plants and prey (over the quality of resources) determine an individual's feeding class and that feeding phenotypes are often the product of convergent evolution of emergent trait complexes under specific environmental conditions. Foraging outcomes appear to be consequences of degree and type of phenotypic specialization for plant and animal prey, navigation and exploitation of the habitat, reproduction, and interactions with other individuals in a heterogeneous environment. Omnivory should not be treated as a fixed strategy, but instead a pattern of phenotypic expression, emerging from diverse genetic sources and coevolving across a range of ecological contexts.

## Introduction

An individual's ability to obtain and assimilate a single food resource often trades off against its ability to use alternate resources. Theory predicts that when given a choice of resources this trade-off should disfavor the evolution of generalist feeding strategies, such as omnivory (e.g., Pérez-Barbería and Gordon 1999; Birdsey et al. 2004; Perry and Roitberg 2005; Evans et al. 2007). Omnivores are generally defined as animals that feed on two or more trophic levels (Pimm and Lawton 1978) or, more specifically, on both plants and animal prey (Lincoln et al. 1998; Coll and Guershon 2002). These latter omnivores are extremely interesting from a functional and evolutionary perspective because differences between plants and animals force trade-offs in the traits required to feed on these distinct resources (Roitberg et al. 2005). For instance, an individual that has the enzymes necessary for breaking down proteins in animal prey generally does not also have all the enzymes capable of digesting plant matter (Agusti and Cohen 2000).

**Figure 1:**
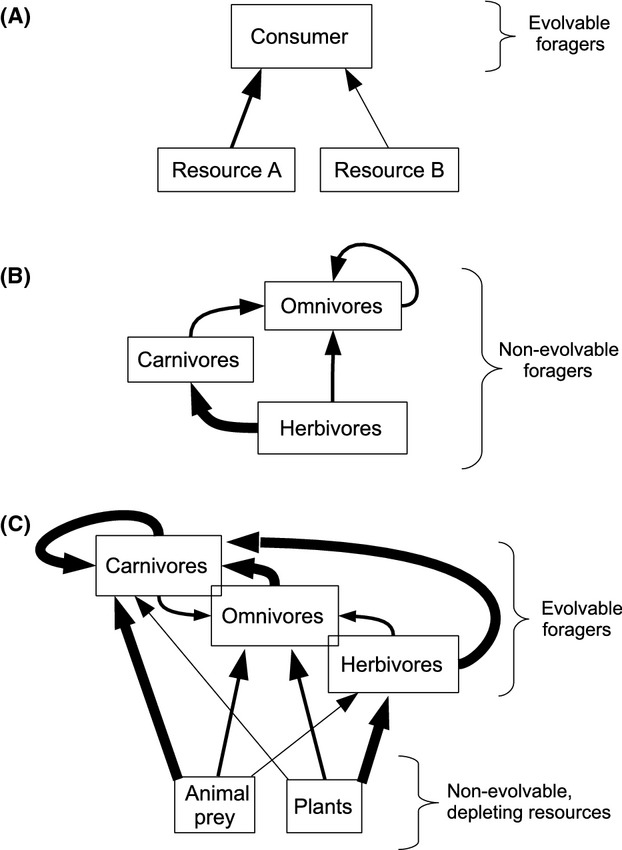
Comparison of three modeling approaches used to understand the evolution of foraging strategies: (A) frequency-dependent specialist–generalist framework; (B) density-dependent trophic omnivory framework (e.g., Pimm and Lawton 1978); (C) our adaptive frequency-dependent phenotypic approach, which allows for the emergence of trophic structure. Arrows indicate direction and relative magnitude of energy transfer between tropic levels or feeding classes.

Analytical approaches have produced a variety of predictions about the prevalence and coexistence of different feeding strategies. Classic optimal foraging theory (Charnov 1976; Krebs and Davies 1993) predicts that if the economically optimal resource is abundant, then specializing on that resource is preferred, whereas if the optimal resource is scarce, then individuals should behave as generalists. From a population dynamics approach, stable coexistence between omnivores and specialists should be rare due to competitive exclusion (Polis et al. 1989; Polis and Holt 1992). However, these studies did not include the ability of feeding strategies to evolve. In examining one predator–two prey coevolution, the evolutionarily stable strategy (ESS) is a generalist strategy when the trade-off for adapting to one resource over the alternate resource was small (see Fig. 1A). Conversely, if trade-offs are large, the ESS may consist of two specialists (Brown and Vincent 1992; Ackermann and Doebeli 2004). Studies integrating these various approaches have yielded insights into the mechanisms promoting coexistence of multiple feeding strategies (e.g., spatial and temporal heterogeneity, adaptive behavior, population structure). We elaborate on such mechanisms as they relate to our model in the Discussion below.

Within a community context, the deceptively simple definitions of feeding classes (e.g., carnivore, omnivore, herbivore, as in Fig. 1B) belies the complexity underpinning the evolution of foraging phenotypes. An individual'vs feeding strategy should comprise not only traits directly associated with utilizing plant and animal prey resources, but also factors that influence their ability to locate and compete for these resources. Thus, in addition to traits influencing the acquisition and assimilation of plant and prey tissues, which we call the “nutritional” traits, we should also consider “non-nutritional” traits.

This study investigates how an individual's suite of evolvable traits (i.e., its strategy), the strategies of others, and the environment might interact to produce feeding phenotypes (i.e., realized diets). Feeding phenotype is an outcome of multivariate (i.e., multidimensional) frequency-dependent processes in a heterogeneous environment. This investigation attempts to address four questions. First, What features of the environment will favor the presence and prevalence of different feeding classes – particularly omnivores – in communities? Second, What combinations of behavioral and physiological traits characterize a feeding phenotype? Third, How do traits differ within and between feeding phenotypes in different environments? Finally, Can we predict an individual's diet based on its intrinsic strategy and the environment?

To answer these questions, we used an evolutionary game-theoretic simulation model to evaluate the success of different feeding strategies under different environmental conditions. Simpler analytical models become intractable when dealing with large sets of possible feeding classes (e.g., Levine 1976; Van Baalen et al. 2001; Grant et al. 2002; Diehl 2003). The simulation approach used here allows a large number of feeding strategies to compete against one another in both frequency-and density-dependent ways (summarized in Fig. 1C).

## The Model

We used an individual-based, evolutionary simulation model, referred to as a genetic algorithm (GA). These are numeric optimization techniques that operate by means analogous to natural selection; they are useful when studying systems that are analytically intractable, due to the large number of strategies (Goldberg 1989; Sumida et al. 1990; Forrest 1993; Axelrod 1997). GAs evaluate the performance of individual strategies while generating new strategies, preferentially propagating successful ones. The strategies that persist at the end of a simulation represent the strategies with the highest success (fitness) in a given environment. They allow traits to evolve independently, and therefore, any commonly occurring combinations of trait values suggest the formation of trait complexes. Although GAs are inspired by and borrow terminology from evolutionary biology, we emphasize that we are only optimizing between different strategies and we are not implying the model accurately represents organic evolution (see Perry and Roitberg 2005); that is, GAs employ the phenotypic gambit (Grafen 1984) in a game context.

The simulations consisted of foraging animals with discrete nonoverlapping generations, and therefore, model dynamics were divided into within-and between-generation components. Within a generation, copies of strategies acted as individuals that interacted with one another and their environment to accumulate fitness. Between generations, the fitness of strategies was evaluated and new strategies were produced based on the fitness of the preceding generation. We summarize the general process (see Figure S1) and provide specific model details, such as parameter values, in the Data S1. More general details are provided below.

### Characterization of the environment

Simulations took place in a spatially explicit, torus-shaped world consisting of a grid of cells (*X*_MAX_ × *Y*_MAX_). Plants and nonforaging animal prey were distributed randomly throughout the environment, but were limited to a maximum number of each per cell, as well as in total for the environment. These two resources are described by their absolute resource quality (*φ*), which is the energy content of a particular resource item, and absolute resource availability (*θ*), which is the number of resource items in the environment. We also recorded the relative resource availability, which is the ratio of plant to prey items in the environment (*ω*). We initialized the forager population using *N* individuals, each with randomly generated starting locations.

### Characterization of a strategy/individual

Each individual in the population was assigned a strategy using the following seven traits: acquisition of prey (*A*_pr_), acquisition of plants (*A*_pl_), assimilation of prey (*B*_pr_), assimilation of plants (*B*_pl_), aggression (*Z*), offspring size (*R*), and mobility (*D*). Specific trait values are denoted as *α*_pr_, *α*_pl_, *β*_pr_, *β*_pl_, *ζ*,*ρ*, and *δ*, respectively. We consider this the minimum set of evolvable traits to allow for an organism to utilize resources, interact with its environment (including other individuals), and reproduce. Each of the seven traits was characterized as follows. Acquisition of plant and prey food sources (*A*_pr_ and *A*_pl_) is defined as the probability of successfully seeking out and acquiring each resource. Assimilation of each resource type (*B*_pr_ and *B*_pl_) is characterized as the proportion of total energy assimilated from that resource (i.e.*,* conversion efficiency). Aggression (*Z*) is the probability of engaging in a competitive interaction with another individual. The trait for offspring size (*R*) assumes individuals may produce many small offspring or fewer large offspring*,* as each individual has a finite pool of energy to allocate among offspring (Smith and Fretwell 1974). Finally, mobility (*D*) determines the probability of leaving an individual's current location.

### Evaluation of fitness

The model's fitness metric is energy, which is assumed to be proportional to body size (*x*). This approach implicitly addresses the general fitness benefits of both energy storage (for metabolic maintenance and reproduction) and larger size (which generally yields greater energy acquisition and fecundity) (Blanckenhorn 2000; Sutherland et al. 2000), without explicitly considering trade-offs among different life-history strategies. Our aim was to reflect a general accumulation of resources without explicitly addressing the complexities of allocation to either body structure or energy storage tissues. Thus, size provides benefits for food acquisition and competitive interactions (i.e.*,* structural size) as well as acts as a metric of energy storage (i.e.*,* storage of reserves).

#### Within-generation dynamics

The model consists of populations of evolvable foraging individuals that consume nonevolvable plants and nonforaging, nonevolvable animal prey (Fig. 1). Within a generation – over the course of *T* time steps – individuals may exploit plant and prey resources, compete with and exploit each other, grow, and move between cells in the environment.

##### Resource exploitation and competition

In a cell, resource exploitation depends on the presence of resources in the current cell, the presence of other individuals, and the individuals' strategies. During each time step, an individual may have the opportunity to have an aggressive interaction with another individual and then consume, if present, both food resources within the cell (Fig. 1C); scramble competition occurs over these resources. Interactions occur between randomly selected pairs of individuals. Only one interaction per individual is allowed. The probability of the members of a pair interacting is determined by the average of the two individuals' *ζ* values, and the outcomes of interactions is determined by the *ζ* values of the individuals as well as their relative sizes, such that individuals with higher *ζ* and *x* are more likely to win. The probability of individual one (1) winning the size-dependent aggressive interaction against individual two (2) is:


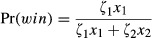
(1)

The outcome of this interaction has two effects: (1) The “winner” gains the opportunity to exploit the patch before the “loser” (i.e., exploitative competition), and (2) the winner also gains a proportion (*λ*) of the loser's fitness, reducing the fitness of the loser. The fitness gained by the winner of the interaction is modulated by the winner's conversion efficiency of prey (*β*_pr_), such that high *β*_pr_ values result in gaining a larger proportion of the loser's fitness. This interaction allows trophic structure to emerge among foragers (Fig. 1C), allowing carnivores or omnivores to consume herbivores as well as one another. Although this may seem odd in a strict biological sense, from a modeling perspective this is justified, because we are ultimately interested in the cumulative fitness of a strategy rather than the individual. Strategies more vulnerable to this form of competition (i.e., intraguild predation) suffered reduced fitness, but were not eliminated based solely on the outcome of one interaction. Additionally, this helps ensure a constant population size, thus avoiding possible confounding effects of population dynamics.

Additionally, plant feeding was contingent on the individual's ability to acquire the resource (*A*_pl_) and to assimilate plant material (*B*_pl_). The maximum energy yielded from plants is *φ*_pl_, but the actual amount of energy they gain depended on their *α*_pl_ and *β*_pl_ values. Likewise, prey feeding was contingent on the individual's *α*_pr_ and *β*_pr_. The maximum energy yielded from prey is *φ*_pr_, which we assumed was proportionately larger than the energy yielded from plants, by a ratio *ξ* (i.e.*, φ*_pr_ = *ξ φ*_pl_; *ξ* > 1).

At the end of each time step, plant and prey resources in each cell are replenished to their initial levels.

##### Growth

Growth of each individual was calculated based on the amount of energy gained during the feeding opportunities at each time step, and offset by a size-dependent basal metabolic cost as well as three trait-mediated costs. Basal metabolic cost was a proportion (*c*_*x*_) of the individual's current size. Trait-mediated costs are dependent on the individual's size. Individuals pay a cost for being aggressive, based on their *ζ*, such that *ψ*_*G*_ = *c*_*G*_ *×* *ζ* × *x*. In evaluating the costs of both acquisition (*ψ*_*A*_) and assimilation (*ψ*_*D*_) of each resource type, we assumed a trade-off: It becomes increasingly expensive to maintain high trait values of both *A*_pr_ and *A*_pl_, or *B*_pr_ and *B*_pl_. Thus, the costs associated with maintaining high trait values are represented as quadratic functions where


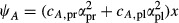
(2a)


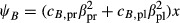
(2b)

with *c*_*i,j*_ representing the proportional cost of maintaining trait *i* for resource *j*.

##### Movement

Movement between cells could occur within each time step and was based on an individual's probability of moving, which was determined by their *δ*. The direction of movement was random, with equal probability of moving into any of the cells within the Moore neighborhood.

#### Between-generation dynamics

Between-generation processes were divided into two components: (1) evaluation of performance and selection, and (2) strategy propagation and variation.

##### Evaluation of performance and selection

More fit strategies were more likely to be selected to propagate. At the end of each generation, the cumulative fitness from all individuals of the same strategy is summed, and the strategy's cumulative fitness is then represented relative to the fitness of all other strategies. Thus, for a given strategy, *i*, fitness [*F*_*i*_(*x*)] was based on the total size (*x*) of all individuals of that strategy:


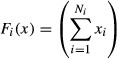
(3)

These proportions correspond to the weighted probabilities of selecting each of the strategies during the production of the next generation.

##### Strategy propagation and variation

To explore the strategy space, we used processes analogous to mutation and recombination of chromosomes to introduce additional variation. Mutation occurred randomly at each bit along the string describing the strategy at some probability (*μ*). Recombination occurred between two selected parental strategies at some rate (*ς*) at a random position along the string describing the strategy. Multiple mutation events were possible, but we allowed a maximum of one recombination event per pairing. The introduction of variability in strategies via these pathways ensures that regions of solution space around fitness optima are explored (Goldberg 1989). Note that the “mutation” and “crossover” rates employed in GAs rarely take values that occur in living biological systems, but rather are chosen to thoroughly investigate the solution space, while searching for globally optimal strategies.

#### Simulation convergence criteria

Each simulation was run until the model had converged onto a stable total population fitness value.

### Classifying feeding classes

Of the individuals present in the final generation, we measured the proportion of plant items relative to prey items in its diet, which is the expression of the strategy of each individual (i.e., its realized diet). We refer to this as an individual's feeding phenotype (*η*). Using *η*, we classified the continuum of feeding phenotypes into five ordinal categories, which we refer to as feeding class: “carnivores” (0.0 ≤ *η *≤ 0.02); “carnivorous omnivores” (0.02 < *η* ≤ 0.34); “omnivores” (0.34 < *η *≤ 0.66); “herbivorous omnivores” (0.66 < *η* ≤ 0.98); and “herbivores” (0.98 < *η *≤ 1.0). We compare differences in trait values between feeding classes.

## Methods

We initialized simulations with a population size (*N*) of 1000 individuals, each with a random strategy. The simulation environment is a 500 × 500 (*X*_MAX_ × *Y*_MAX_) cell torus, with each cell corresponding to a patch. We ran simulations for 100 generations (G), with 100 time steps (T) per generation, and reinitialized population size to 1000 individuals at the beginning of each generation. We varied three environmental factors: absolute resource quality (*φ*; 9 levels), absolute resource availability (*θ*; 9 levels), and the relative resource availability (*ω*; 9 levels). Together, absolute resource quality and absolute resource availability determine environmental productivity. Relative resource availability determines the composition of the environment. The combinations of environmental factors that we considered are represented by a complete three-factor experimental design (Table 1), and simulations were replicated three times per treatment combination. Replication is used not for statistical power, but to ensure that the solutions converge upon a global optimum instead of on local optima. All remaining parameters that were not examined, including relative resource quality (*ξ*), were held constant (Table S1). We also performed additional simulations keeping population size constant but changing the size of the environment (i.e., changed *X*_MAX_ and *Y*_MAX_), thus changing the population density (see Table S1). Across these simulations, we examined the trait values of each individual, and the success of that individual [*F*(*x*)] in a given environment.

**Table 1:** Factorial design of the range of environmental parameters explored in the simulations

Factor	Parameter	Range of values
Absolute resource availability	*θ*_pl_ *θ*_pr_	0.05–0.45 *θ*_pl_/*ω*
Absolute resource quality	*φ*_pl_ *φ*_pr_	20–100 *ξ φ*_pl_
Relative resource availability	*ω*	1–40
Relative resource quality	*ξ*	10

Given the explicit structure of the experimental design, we used standard statistical approaches to analyze the model output, with four primary aims: (1) We evaluated how the frequency of feeding phenotypes within a community changes in relation to environmental conditions; (2) we identified emergent trait complexes within the seven traits; (3) we considered the role of the environment in generating emergent trait complexes; and (4) we developed a statistical model to predict feeding phenotype (*η*) using the array of environmental conditions and the seven traits that characterized each strategy.

### Community composition

We used ordinal logistic regression (cumulative logit) models to evaluate how the frequency of feeding phenotype changed in relation to the three environmental factors (higher-order environmental terms were included as orthogonal polynomials). We used a goodness-of-fit test (*χ*^2^) against a full model to select the simplest model.

### Identification of emergent trait complexes

We used principal components analysis (PCA) based on the correlation matrix to identify and describe emergent trait complexes and retained axes with eigenvalues > 1. PCA is used to identify orthogonal groupings of factors and their relative importance. It is frequently used as a variable reduction technique (Jackson 1991).

### Characterization of feeding classes

We evaluated the role of the environment in generating these emergent trait complexes using a weighted multivariate generalized linear mixed model with ordinal feeding classes. Strategy was the response variable, represented by scores from the retained principal component (PC) axes. Environmental conditions (*φ*,*θ*, and *ω*) were included as covariates, with their second-and third-order terms included as orthogonal polynomials. Simulation replicates were included as a random variable. Higher-order terms were included because the effect of environment on phenotype was expected to be complex. To examine the effects of a single environmental factor on trait values, we held all of the other environmental factors constant at their means and compared adjusted mean trait values. We weighted strategies by fitness because strategies with higher fitness make a larger contribution to trait means. We performed sequential contrasts to compare PC scores and log-transformed mean size at the end of generation [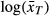] between feeding classes.

### Predicting feeding phenotypes

To predict feeding phenotype (*η*) based on traits and environmental factors, we used a weighted linear mixed effects model. We logit-transformed the dependent variable (*η*) prior to analysis. Here, the independent variables were the three retained PC axes, the three environmental factors, and their higher-order terms (included as mean-centered polynomials). Higher-order terms were included to allow for complex relationship between phenotype and environment. Simulation replicates were included as a random variable. As above, we weighted strategies by their fitness. Due to computational limitations, we did not include interaction terms. We used AIC scores to compare reduced models and selected the model with the lowest AIC score. We used sums of squares to interpret the relative importance of model terms. We used bootstrap analysis to demonstrate that the predictive model is robust to missing data. To do this, we randomly subsampled output from 60% of the treatment combinations to fit the model and then performed correlation (*r*_*p*_) analyses between the predicted values and the observed values from the remaining 40% of the treatment combinations. This procedure was repeated 100 times to generate a distribution of correlation coefficients.

Analyses were performed using the R programming language and environment version 2.15.3 (R Development Core Team 2013), using the effects, lme4, and VGAM packages.

## Results

### Community composition

**Figure 2:**
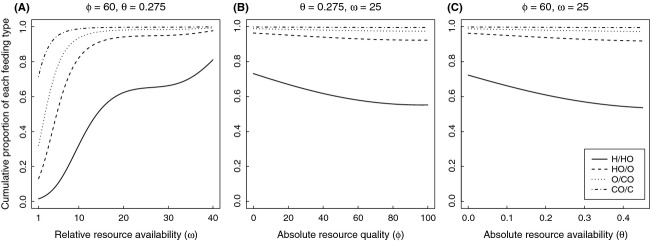
Cumulative proportion of each feeding class across each environmental gradient: (A) relative resource quality (*ω*); (B) absolute resource quality (*φ* ≡ *φ*_pl_ = *φ*_pr_/*ξ*); and (C) absolute resource availability 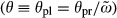. Solid lines denote boundary between herbivores (H) and herbivorous omnivores (HO). Dashed lines denote boundary between herbivorous omnivores (HO) and omnivores (O). Dotted lines denote boundaries between omnivores (O) and carnivorous omnivores (CO). Dash-dotted lines denote boundaries between carnivorous omnivores (CO) and carnivores (C).

The best ordinal logistic regression model describing how frequency of feeding phenotypes changed within a community included the first-and second-order terms for each of the environmental factors, plus the third-order term for relative resource availability (*χ*^2^ = 91917, df = 7, *P* ≪ 0.001; Table 2). Evolutionarily stable communities were strongly polytypic: They always included a range of feeding phenotypes, from omnivores to herbivores. Omnivores were present in all communities; however, herbivores were the dominant feeding phenotype in most environments (Fig. 2).

**Table 2:** Ordinal logistic regression model (cumulative logistic regression) describing the proportion of each feeding type (γ) across environmental gradients: relative resource availability (*ω*), absolute resource quality (*φ*), and absolute resource availability (*θ*). Higher-order terms are specified through orthogonal polynomials

Factor	Estimate	SE	*t*-value	*P*-value
Intercept1	−0.328	0.004	−88.0	<0.001
Intercept2	1.962	0.005	365.8	<0.001
Intercept3	3.098	0.007	459.9	<0.001
Intercept4	4.755	0.009	533.1	<0.001
*φ*_pl_	−4.417	0.077	−57.1	<0.001
*φ*_pl_^*2*^	1.235	0.078	15.8	<0.001
*ω*	40.517	0.098	412.3	<0.001
*ω*^*2*^	−18.406	0.091	−202.3	<0.001
*ω*^*3*^	10.705	0.083	128.8	<0.001
*θ*_pl_	−5.908	0.086	−68.9	<0.001
*θ*_pl_^*2*^	1.286	0.082	15.6	<0.001

Log likelihood = −36321.8; df = 2905.

*χ*^2^ = 91917; df = 7; *P*-value = <0.001.

**Figure 3:**
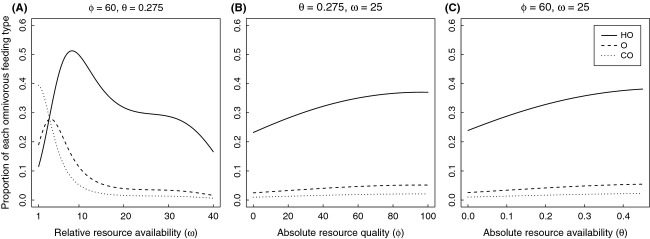
Proportion of omnivorous feeding classes across each environmental gradient: (A) relative resource quality (*ω*); (B) absolute resource quality (*φ* ≡ *φ*_pl_ = *φ*_pr_/*ξ*); and (C) absolute resource availability 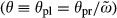. Solid, dashed, and dotted lines denote herbivorous omnivores (HO), omnivores (O), and carnivorous omnivores (CO), respectively.

Relative resource availability (*ω*) exerted a much stronger effect on feeding phenotype frequencies than either absolute resource quality (*φ*) or absolute resource availability (*θ*; Figs. 2, 3). Community composition shifted strongly between low and intermediate *ω* levels, although from intermediate and high *ω* levels, the community was dominated by herbivores and omnivorous herbivores (Fig. 3A). At low *ω* levels omnivores and carnivorous omnivores, dominated community composition (Fig. 3A). Focusing specifically on the influence of *ω* on the three omnivore classes (Fig. 3A), the prevalence of herbivorous omnivores rose quickly in response to *ω*, reaching maximum frequencies at relatively low *ω* values; thereafter, herbivorous omnivores decreased quickly until intermediate *ω* values were reached and they continued to decline slowly. Omnivores exhibited an initial increase with *ω* and then decreased relatively quickly. Carnivorous omnivores had maximal frequencies at low *ω* values and decreased to near-zero frequencies at relatively low *ω* values.

In contrast, absolute resource quality (*φ*) and absolute resource availability (*θ*) had a less pronounced effect on community composition. The prevalence of all omnivore classes was positively associated with both *φ* and *θ* (Fig. 3B and C), while the opposite was true for herbivores (Fig. 2B and C). Like omnivores, the prevalence of carnivores increased, though to a much lesser extent, with increasing *φ* and *θ* (Fig. 2B and C).

These results are robust to alternate interpretations of individuals' phenotype describing its feeding class. We obtain qualitatively similar results using alternate cutoff points for the bins of each of our five feeding classes (e.g., “carnivores” 0.0 ≤ *η *≤ 0. 20; “carnivorous omnivores” 0.20 < *η *≤ 0.40; “omnivores” 0.40 < *η *≤ 0.60; “herbivorous omnivores” 0.60 < *η *≤ 0.80; and “herbivores” 0.80 < *η *≤ 1.0).

### Identification of emergent trait complexes

Three axes were retained from a PCA on the seven traits and account for 55% of the variation in the traits (Table 3). Acquisition and assimilation of prey (*A*_pr_ and *B*_pr_, respectively) loaded positively, while acquisition and assimilation of plants (*A*_pl_ and *B*_pl_, respectively) loaded negatively, on PC1. Mobility (*D*) loaded positively and offspring size (*R*) negatively on PC2. Aggression (*Z*) dominates PC3 and loaded positively with the residual nutritional traits, whereas offspring size loaded negatively.

**Table 3:** Principal components analysis of trait values based on correlation matrix. Axes with eigenvalues > 1 were retained. Loadings of dominant traits are bolded

	PC1	PC2	PC3
Importance of Components:			
Eigenvalue	1.33	1.03	1.01
Proportion of variance explained	0.25	0.15	0.15
Cumulative proportion of variance explained	0.25	0.40	0.55
Loadings			
*A*_pr_	**0.39**	−0.03	0.17
*A*_pl_	−**0.59**	−0.06	0.10
*B*_pr_	**0.39**	0.01	0.15
*B*_pl_	−**0.59**	−0.04	0.11
*Z*	0.01	−0.16	**0.94**
*R*	0.07	−**0.67**	−0.20
*D*	−0.01	**0.72**	0.05

### Characterization of feeding classes

**Figure 4:**
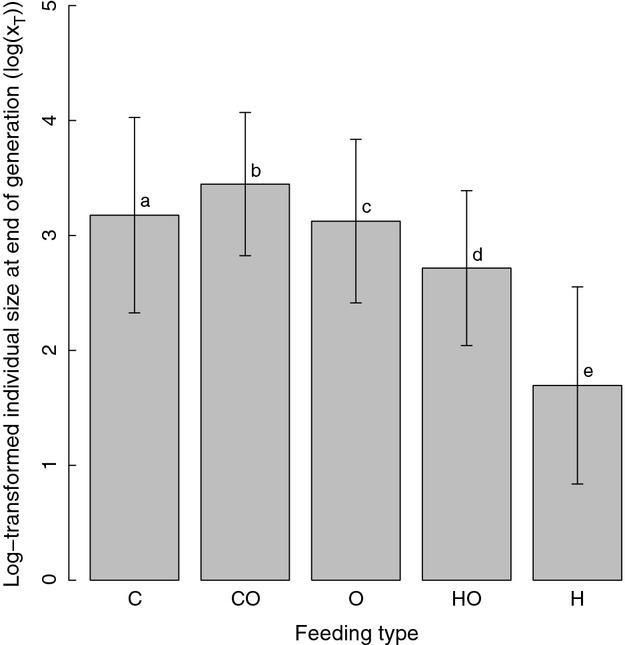
Log-transformed mean individual size at the end of a generation [log(*x*_*T*_)] for each feeding class. Error bars indicate log standard deviations. Letters indicate differences between feeding classes.

Carnivorous omnivores had the largest log-transformed mean size at the end of the generation [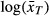] and herbivores the smallest (Fig. 4); however, there was a wide range of sizes [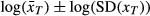]: 3.18 ± 0.87; 3.46 ± 0.63; 3.14 ± 0.72; 2.73 ± 0.68; 1.71 ± 0.86). A linear mixed effects model demonstrated significant differences in size among the ordinal feeding classes (approx. *F*_4,1021943_ = 201 381, *P* ≪ 0.001). Contrasts demonstrated that feeding classes differed sequentially for log(*x*_*T*_) (all |*t*| > 143.7, all *P* ≪ 0.001).

We considered the influence of the environment in generating the emergent trait complexes represented by the three retained PC axes. Using a weighted multivariate generalized linear mixed model, we demonstrated a strong overall effect of differences between feeding phenotypes on all seven PC axes (approx. *χ*^2^_49,1021943_ = 1.2 × 10^6^, *P* < 0.001); however, this result was driven primarily by PC1 (Table 4; approx. *F*_4,1021943_ = 9.9 × 10^8^, *P* ≪ 0.001). PC2 and PC3 also varied, but the explanatory power was weaker than for PC1 (PC2: approx. *F*_4,1021943_ = 1.0 × 10^7^, *p* ≪ 0.001; PC3: approx. *F*_4,1021943_ = 1.5 × 10^7^, *P* ≪ 0.001). Again, contrasts demonstrated that feeding classes differed sequentially along the continuum of feeding phenotypes for each PC axis (all *|t|* > 26, all *P* ≪ 0.001).

**Table 4:** Multiple generalized linear mixed effects models (multivariate GLMM results in text) of retained principal component (PC) axes for each feeding type (γ), using resource availability (*ω*), absolute resource quality (*φ*), and absolute resource availability (*θ*) as predictor variables

		PC1			PC2			PC3		
	df	Sum of squares	*F*-value	*P*-value	Sum of squares	*F*-value	*P*-value	Sum of squares	*F*-value	*P*-value
*γ*	4	1.5322E+09	9.8997E+08	<0.001	3.0857E+07	1.0083E+07	<0.001	4.6374E+07	1.4623E+07	<0.001
*φ*_pl_	1	3.0013E+07	7.7568E+07	<0.001	1.1781E+05	1.5398E+05	<0.001	3.7036E+04	4.6713E+04	<0.001
*Ω*	1	8.1142E+07	2.0971E+08	<0.001	4.8913E+05	6.3932E+05	<0.001	2.1758E+06	2.7443E+06	<0.001
*θ*_pl_	1	8.7554E+05	2.2628E+06	<0.001	4.8180E+03	6.2973E+03	<0.001	3.3894E+05	4.2749E+05	<0.001
*φ*_pl_^*2*^	1	3.7002E+06	9.5630E+06	<0.001	8.5910E+03	1.1229E+04	<0.001	1.2444E+05	1.5696E+05	<0.001
*φ*_pl_^*3*^	1	1.9740E+03	5.1017E+03	<0.001	6.7285E+06	8.7946E+06	<0.001	1.8304E+04	2.3087E+04	<0.001
*ω*^*2*^	1	4.2877E+06	1.1081E+07	<0.001	1.0009E+07	1.3083E+07	<0.001	6.6895E+05	8.4373E+05	<0.001
*ω*^*3*^	1	1.9294E+07	4.9864E+07	<0.001	6.7285E+06	8.7946E+06	<0.001	8.2307E+05	1.0381E+06	<0.001
*θ*_pl_^*2*^	1	3.8441E+05	9.9349E+05	<0.001	8.9191E+05	1.1658E+06	<0.001	9.6558E+05	1.2179E+06	<0.001
*θ*_pl_^*3*^	1	1.3734E+05	3.5496E+05	<0.001	6.9775E+04	9.1199E+04	<0.001	1.1400E+02	1.4374E+02	<0.001
*γ: φ*_pl_	4	4.6140E+07	2.9812E+07	<0.001	6.4065E+05	2.0934E+05	<0.001	1.4870E+06	4.6887E+05	<0.001
*γ: ω*	4	5.1135E+06	3.3039E+06	<0.001	2.0287E+06	6.6291E+05	<0.001	2.1965E+05	6.9260E+04	<0.001
*γ: θ*_pl_	4	1.2002E+06	7.7544E+05	<0.001	4.2979E+06	1.4044E+06	<0.001	3.1663E+05	9.9838E+04	<0.001
*γ: φ*_pl_^*2*^	4	1.1652E+06	7.5285E+05	<0.001	5.5102E+04	1.8005E+04	<0.001	2.6793E+05	8.4483E+04	<0.001
*γ: φ*_pl_^*3*^	4	1.8907E+05	1.2216E+05	<0.001	1.0313E+05	3.3698E+04	<0.001	1.6575E+05	5.2264E+04	<0.001
*γ: ω*^*2*^	4	5.2508E+06	2.9035E+06	<0.001	9.5286E+05	3.1136E+05	<0.001	1.3752E+05	4.3364E+04	<0.001
*γ: ω*^*3*^	4	4.4937E+06	7.7544E+05	<0.001	1.2314E+06	4.0239E+05	<0.001	6.2748E+05	1.9786E+05	<0.001
*γ: θ*_pl_^*2*^	4	3.3366E+05	2.1558E+05	<0.001	1.5289E+06	4.9959E+05	<0.001	5.1468E+04	1.6229E+04	<0.001
*γ: θ*_pl_^*3*^	4	4.1601E+04	2.6879E+04	<0.001	3.0727E+04	1.0040E+04	<0.001	2.2768E+05	7.1792E+04	<0.001
Residuals	477666	1.7359E+09			6.6775E+07			5.5027E+07		

**Figure 5:**
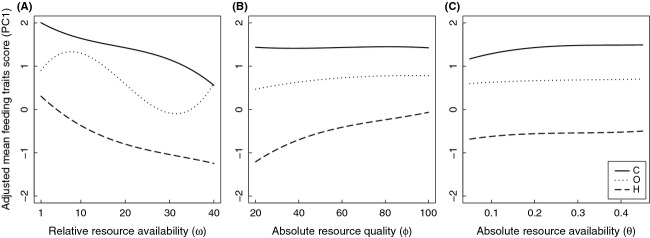
Effects plots from a weighted multivariate generalized linear mixed effects model show adjusted mean feeding trait score (*PC1*) across each environmental gradient: (A) relative resource quality, *ω*; (B) absolute resource quality (*φ* ≡ *φ*_pl_ = *φ*_pr_/*ξ*); and (C) absolute resource availability (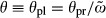). Along each gradient, the other two factors are held constant at their means. Adjusted mean feeding traits score (*PC1*) describes *A*_*i*_ and *B*_*i*,_ such that higher *PC1* values reflect higher *α*_pr_ and *β*_pr_ and lower *α*_pl_ and *β*_pl_. Solid lines represent carnivores (C), dotted lines represent omnivores (O), and dashed lines represent herbivores (H). PC2 and PC3 scores were only weakly influenced by environment.

Across all the three environmental gradients, the most dramatic differences among feeding class occurred with PC1 scores (Fig. 5; results for PC2 and PC3 not shown). However, the optimal values for the non-nutritional traits – aggression (*Z*), offspring size (*R*), and mobility (*D*) – did not change dramatically with changes in the environment or between feeding class, and all converged on low values (mean ± SD: *ζ *= 0.24 ± 0.30; *ρ *= 12 ± 4.4; *δ *= 0.35 ± 0.25). In the following analyses, we only consider PC1 (the nutritional traits) because of the weak influence of environmental conditions on shaping PC2 and PC3 scores.

For each environmental factor, the three omnivore classes had PC1 scores intermediate to carnivores and herbivores, which had the highest and lowest values, respectively. The high PC1 scores of carnivores reflects their higher trait values of acquisition and assimilation of prey (*A*_pr_ and *B*_pr_), whereas the low PC1 scores of herbivores reflects their higher trait values of acquisition and assimilation of plants (*A*_pl_ and *B*_pl_). Across all environmental gradients, there was a bias in trait values to higher values of *α*_pr_ and *β*_pr_ than *α*_pl_ and *β*_pl_ (Fig. 5).

As relative resource availability (*ω*) increased (i.e., when the world becomes “greener”)*,* PC1 scores in carnivores and herbivores decreased and fluctuated for omnivores (Fig. 5A). The decrease in PC1 scores indicated that traits associated with feeding on plants increased in all three classes as *ω* increased. Increasing resource quality (*φ*; Fig. 5B) had little effect on the PC1 scores of carnivores, whereas omnivore PC1 scores were smaller in magnitude and increased only slightly with increasing *φ*. Herbivore scores were initially negative and increased substantially with increasing *φ* to positive scores. Changes in absolute resource availability (*θ*) had little effect on PC1 scores for each feeding class (Fig. 5C), with omnivore maintaining moderately low positive scores. Carnivores and herbivores maintained moderately large positive and small negative scores, respectively.

**Figure 6:**
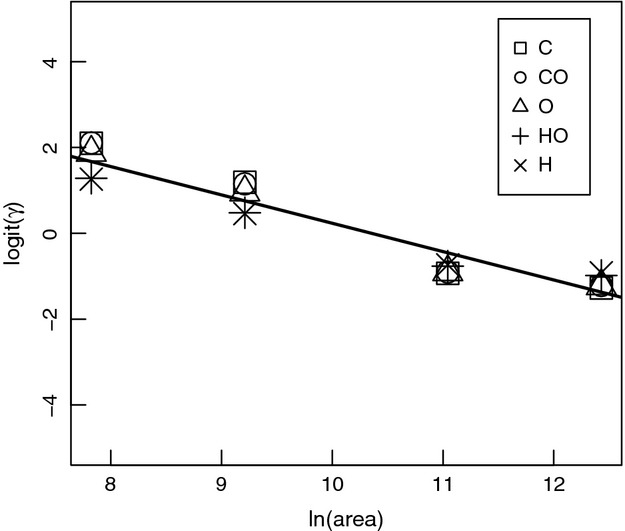
Logit-transformed mean aggression values [logit(γ)] across several world area sizes.

Across a range of world sizes (with population size held constant), we found that aggression (*Z*) was the only trait that varied systematically with changes in population density. Analysis of covariance (ANCOVA) demonstrated that aggression was independent of feeding class (*F*_4,14_ = 0.02, *P *=* *1.0), so we excluded this term from the model. In the reduced model, there was a strong inverse relationship between aggression and density (Fig. 6; adjusted *r*^2^ = 0.92, *F*_1,2_ = 34.97, *P *=* *0.03). All other results were qualitatively consistent across the range of world sizes tested.

### Predicting feeding phenotypes

Environmental conditions and strategy traits were strong predictors of feeding phenotype (*η*). Feeding phenotype was predicted by PCs 1–3, all of the first-, second-, and third-order terms for each of the environmental factors, but none of their interactions (approx. *χ*^2^_15,1021943_ = 976985, *P* ≪ 0.001, Table 5). The abilities to acquire and assimilate prey vs. plants (PC1) were the strongest intrinsic predictor of feeding phenotype (Table 5, sums of squares). Of the environmental conditions, relative resource availability (*ω*) was the most important predictor of feeding phenotype (Table 5). Absolute resource quality (*φ*) and absolute resource availability (*θ*) contributed little in comparison. Bootstrap analysis produced a set of highly correlated *r*_*p*_ values between the observed and fitted data (mean *r*_*p*_ = 0.71, SD = 0.02), demonstrating that our predictive model is robust.

**Table 5:** A linear mixed effects model that predicts feeding phenotype from the set of environmental genotypic factors, using simulation run as a random intercept (SD = 0.001; residual SD = 2.033)

Parameter	Sum of squares	Estimate	SE	*t*-value	*P*-value
Environmental factors					
*ω*	1.0120E+10	1.451E−05	1.451E−05	1519	<0.001
*ω*^*2*^	8.8179E+08	−4.740E−03	4.352E−07	−10981	<0.001
*ω*^*3*^	1.0970E+08	4.352E−07	4.258E−08	5472	<0.001
*φ*_pl_	4.3927E+08	−8.330E−04	4.476E−06	−186	<0.001
*φ*_pl_^*2*^	5.3009E+07	−1.190E−05	1.069E−07	−111	<0.001
*θ*_pl_	1.4034E+07	4.584E−01	9.548E−04	480	<0.001
*θ*_pl_^*2*^	3.0558E+06	−3.863E+00	3.635E−03	−1063	<0.001
*θ*_pl_^*3*^	1.1922E+06	2.243E+01	3.285E−02	683	<0.001
*φ*_pl_^*3*^	1.1910E+03	8.976E−07	3.761E−09	239	<0.001
Genotypic factors					
*PC1*	2.2178E+09	−1.372E+00	5.942E−02	−23094	<0.001
*PC2*	7.2307E+06	−6.574E−02	5.067E−05	−1297	<0.001
*PC3*	1.6450E+06	3.145E−02	4.987E−05	631	<0.001
Intercept		2.066E+00	7.489E−04	2759	<0.001

Overall model: 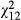
 = 976985; AIC *=* 12 142 669; *P *<* *0.001.

## Discussion

### Community composition

Classic optimal foraging theory (OFT) predicts that the benefit of feeding on more than one prey type should be independent of the ratio between resources and exclusively dependent on the rarity of the most profitable resource (Charnov 1976); however, of the variables we considered, relative resource abundance had the most profound effect on community composition (Figs. 2, 3). Pure carnivores were rare across all simulations, consistent with empirical and theoretical expectations (e.g., Colinvaux 1979; Farlow 1993; Spencer 2000). As plants became more common relative to animal prey, there was an increase in the frequency of herbivores as well as an initial increase in the number of omnivores. As the world became increasingly plant dominated, herbivores continued to increase but omnivore frequency decreased. In contrast, as habitat productivity increased, omnivores became more prevalent and herbivores less so. Thus, increased productivity led to greater abundance of higher trophic levels (e.g., Beveridge et al. 2010). These results suggest that different environmental pressures promoted shifts in feeding mode, from specialist to generalist, leading to the ubiquity of omnivores in our simulations.

Our results support the idea that adaptive omnivorous behavior such as diet expansion in response to changes in enrichment or environmental productivity and competition could lead to increased prevalence of omnivores (e.g., Křivan and Schmitz 2003). In real systems, lower levels of competition may favor generalists over specialists, resulting in prevalence and persistence of omnivores. For example, during periods of prey scarcity with an abundance of plants (i.e., at low to intermediate relative resource availability), prey may become more difficult for carnivores to access in communities as a result of either density-or frequency-dependent processes. This should favor omnivores that can better feed on plants (Lalonde et al. 1999; Singer and Bernays 2003). Thus, omnivory may allow for reduction in competition due to the consumption of alternate resources when density-dependent productivity declines. Omnivory may also evolve as a response to high environmental heterogeneity, which favors the maintenance of diet breadth. Additionally, increasing disparity in the value of plant vs. animal food resources is positively correlated with the persistence of omnivorous feeding phenotypes in communities (Křivan and Diehl 2005). In other diet choice models, omnivory was only a persistent strategy when there was a synergistic effect of eating plant and animal prey (e.g., Bjorndal 1991); however, in our model, omnivores were common without this assumption.

Why then do specialists evolve and coexist with generalists? The coexistence of all feeding classes shows that negative frequency-dependent diet choice promotes diversification into specialist strategies (e.g., Rueffler et al. 2006). Coexistence of multiple strategies is addressed by niche theory (Schoener 1974; Stephen and Tilman 1994; Levine and HilleRisLambers 2009). Here, individuals and their resulting phenotypes are modeled using a multidimensional approach. Instead of simply allowing the evolution of foraging strategies into specialist classes or a generalist class via the evolution of a single trait (e.g., Brown and Vincent 1992; Ma and Levin 2006), our model allowed multiple traits to coevolve. This vastly increases the potential niche space of our virtual world, decreasing niche overlap, thereby promoting coexistence of many feeding classes (Schoener 1974; Stephen and Tilman 1994; Levine and HilleRisLambers 2009). Empirical and theoretical studies have demonstrated the importance of functional traits and trade-offs in driving community dynamics, defining ecological niches, and maintaining biological diversity (Lubchenco and Menge 1978; Fry 1990; Litchman et al. 2007; Henry et al. 2008).

### Identification of emergent trait complexes

Trade-offs within and among nutritional traits may facilitate shifts in feeding mode, particularly toward specialization. We found nutritional traits formed an emergent trait complex, with assimilation and digestion of prey negatively correlated with the assimilation and digestion of plants. This negative correlation highlights the trade-offs caused by physical and biochemical constraints; traits that confer a fitness advantage for one function often simultaneously reduce the ability to perform other functions (Pérez-Barbería and Gordon 1999; Denno and Fagan 2003; Eubanks et al. 2003; Birdsey et al. 2004; O'Grady et al. 2005; Evans et al. 2007; Herrel et al. 2008).

Interestingly, the non-nutritional traits mobility and offspring size traded off and formed a trait complex. Increases in mobility resulted in decreased offspring size (and vice versa). This effect may be a consequence of dispersal in a heterogeneous environment; production of many small, mobile offspring ensures dispersal to exploit more productive patches (e.g., Barlow 1981). Although both traits maintained relatively low values, there was greater variation in mobility than in offspring size. A greater proportion of individuals produced many small offspring with high mobility, rather than larger, less mobile offspring. This result (see Marshall et al. 2008) may have occurred because offspring were dispersed across the environment at the beginning of a generation. This global dispersal of offspring was carried out to prevent local (i.e.*,* within-patch) conditions to driving changes in traits. If, however, offspring from a single individual are laid in a single patch, we would predict a greater effect size for offspring size and mobility due to enhanced local competition. Mobility reduces competition among kin (e.g., Gandon 1999); therefore, relatively low mobility may have evolved as competition with kin was not possible due to dispersal not being local.

Somewhat surprisingly, aggression was maintained at low levels in the simulations. It was also positively correlated with nutritional traits for both prey and plants. Aggression carries implicit direct and indirect costs, such as increased risk of injury or death and energy costs of sustaining increased endocrine hormone (Briffa and Sneddon 2007). From the PCA results, individuals that paid the cost of maintaining high nutritional trait values tended to be aggressive (Table 3). This result suggests that specialists may evolve higher levels of aggression than generalists, which is consistent with empirical studies (e.g., Marchetti 1999; Yonekura et al. 2008).

### Characterization of feeding classes

Dependence of feeding phenotype on environmental conditions is common across taxa (e.g., Bicca-Marques et al. 2009; Joordens et al. 2009; Ortiz-Catedral and Brunton 2009; Paralikidis et al. 2010), and we found that as the environment changed the inherent strategy describing a feeding class shifted: Extrinsic factors shaped the traits that made up different feedings classes.

The most striking differences among feeding classes occurred in the nutritional traits; however, non-nutritional traits also varied among classes. As the number of plants increased relative to the number of animal prey in the environment (i.e., as the world became “greener”), there was a shift toward the maintenance of traits associated with utilizing plant matter. Herbivores maintained high trait values for plant use, and omnivores also shifted toward higher values. Carnivores also shifted toward greater capacity for plant use, but not to the same degree as omnivores (Fig. 5A). This result is consistent with the predictions of diet balancing models, which suggest that in some cases, foraging decisions can depend on the ratio of different resources (Fryxell and Lundberg 1998).

As the quality of animal prey increased, so did the benefit of being able to eat prey. For omnivores, this benefit outweighed the costs of maintaining traits that allowed feeding on multiple trophic levels. Our results suggest that carnivores and omnivores, feeding classes that specialize on the highest quality resource or already feed on multiple trophic levels, are less sensitive than herbivores to changes in the quality of resources (Fig. 5B).

Our results suggest that offspring size is largely insensitive to changes in environmental parameters; however, carnivorous classes grew to larger sizes. In the simulations, initial offspring size was relatively low for all feeding classes, but the average final size of individuals at the end of a generation differed dramatically among feeding classes. Omnivores and carnivores produced larger offspring than herbivores, and individual carnivores (and to a lesser extent individual omnivores) grew to be much larger than the individuals at lower trophic levels, which is consistent with the prediction that carnivores grow larger to exploit their prey (Arim et al. 2010).

Population density of foraging individuals, followed by absolute and relative abundance of resources, had the largest effects on aggression, generating patterns that suggest frequency-dependent interaction. Aggression was positively correlated with density (Fig. 6), yet decreased in all feeding phenotypes as the absolute availability of resources increased. This suggests that as competition for resources declined, maintenance of this expensive trait, aggression, was reduced. At high prey abundance, carnivores and omnivores had low aggression because there was little need to compete for this profitable resource. However, as the ratio shifted to a greater abundance of plants, carnivores and omnivores had to engage in more aggressive interactions in holding prey resources. These results are consistent with studies that have shown that aggressive interactions decline with high levels of resource abundance in a wide range of aquatic and terrestrial organisms, including herbivores (e.g.*,* white tailed deer), omnivores (e.g.*,* cichlids), and carnivores (e.g.*,* hyenas) (Grant et al. 2002; Wachter et al. 2002; Vogel and Janson 2007).

### Predicting feeding phenotypes

Our analysis demonstrates that the phenotype of all feeding classes (including omnivores) is both predictable and explainable based upon the underlying intrinsic abilities (i.e., trait complexes) of an individual and its environment. This is not a trivial prediction, given the potential for complex (frequency-and density-dependent) interactions among feeding classes. Given some distribution of intrinsic trait values that arise as an outcome of an evolutionary process, it is possible to predict the phenotypic distribution across all individuals at the community level within a given environment. Our predictive model shows that aggression, mobility, and parental investment may be used to predict omnivory. This finding is consistent with other studies that have suggested mobility may predict omnivorous strategies (e.g., Rosenheim and Corbett 2003). Despite the complexity of determining a forager's feeding phenotype, our model allowed us to predict a forager's feeding phenotype using nutritional and non-nutritional traits, rather than only using biomechanical constraints (e.g., Osenberg et al. 2004).

Our results suggest that the intrinsic traits of a feeding class in one environmental context may not describe the same feeding class in a different one. Thus, the simple definition of an omnivore based on solely its diet (i.e., as an organism that feeds on both plants and animal prey) belies the intricacies of interactions among the environment, an individual's intrinsic abilities, and its realized diet. Omnivores and the other feeding classes must maintain traits that allow capture and utilization of plants and animal prey in the presence of competitors in a particular environmental context. Furthermore, these traits coevolve in response to the environment.

## Conclusions

Omnivores and other feeding classes can be described by the suite of behavioral and physiological traits that encompass their ability to acquire and assimilate both plant and animal foods. In our evolutionary simulation model, omnivores tended to have nutritional traits that were intermediate in value to carnivores and herbivores, but that were biased toward the acquisition and assimilation of animal prey. This study demonstrates the importance of considering omnivory in a community context – the other organisms an omnivore interacts with and the resources available to them. We found that while the heritable basis of a feeding strategy may differ among environments, the expression of that strategy can remain the same. Thus, ecological context acts along with intrinsic traits act to determine the realized diets of animals. In our simulations, omnivores were ubiquitous across all environmental conditions, although for the most part, they were not the dominant feeding class. Omnivores were most prevalent when the ratio between plants and animal prey numbers was low, and to a lesser degree, when habitat productivity was high. These outcomes were likely consequences of lower competition and a relief from trade-offs under a generalist strategy.

## Supporting Information

Figure S1 Schematic representation of the evolutionary simulation model.

Data S1 Model description.

**Table S1.** Description of symbols used. **Table S2.** Summary of descriptions and values corresponding to each trait.
