# Supplementary material for: On the evolution of omnivory in a community context
Source: Ecol Evol. 2013 Dec 29;4(3):251–65. doi: 10.1002/ece3.923 (PMC3925427; doi:10.1002/ece3.923)
Supplement: Data S1 — Model description. [file ece30004-0251-sd2.docx]

# DATA S1

## Model description

### Characterization of a strategy/individual

Each strategy was encoded on a binary string: *Z* occupies two bits, and the other traits each occupy three bits; for a total string length of 20 bits. Thus, there are 2^20^ (1,048,576) unique strategies, highlighting the requirement of a robust search algorithm for evolutionary stable strategies.

## Evaluation of fitness

### Strategy propagation and variation

Furthermore, we used an assortative pairing routine, which allowed a simulation to converge on a solution quickly by minimizing the possibility that the hybrid offspring of two different yet successful strategies exhibit reduced fitness which can destabilize the convergence on a solution (Goldberg 1989). For each selected pair, consisting of strategy *S*_1_ and strategy *S*_2_, we summed the ratios of the raw trait values (0–7) for each trait (*j*), where *J* is the maximum trait value and *T­_j_* is the total number of trait values for trait *j*, to determine a difference score, *κ*, as follows:

$\kappa=\sum_{j=1}^{J} \frac{\left|S_{1,j}-S_{2,j}\right|}{T_{j}}$ (eq. S1)

The probability that strategies *S*_1_ and *S*_2_ do assort is a decreasing linear function of the difference between strategies *S*_1_ and *S*_2_:

$\Pr(assort)=1-\frac{\kappa}{J}$ (eq. S2)

During a pairing event, either the two ‘parent’ strategies make identical copies of themselves (if neither mutation nor crossover occur), or variant ‘offspring’ strategies are produced (if mutation or crossover occur). Using the resultant strategies as templates, each pairing resulted in two groups of offspring, where the body size of each group was defined by the parents’ *ρ*-value, and the potential number of offspring was determined by the ratio of respective average strategy fitness for each parent to *ρ*. The number of offspring from all pairing events was then scaled to the total population size of *N*. We scaled deterministically using the integer portion and sorted the remainders (fraction portion) from highest to lowest (Goldberg, 1989). We then assigned the remaining individuals (to reach *N*) based on the remainders, sequentially assigning individuals to the strategies with the highest fitness first.
