# Supplementary material for: On the evolution of omnivory in a community context
Source: Ecol Evol. 2013 Dec 29;4(3):251–65. doi: 10.1002/ece3.923 (PMC3925427; doi:10.1002/ece3.923)
Supplement: Supplementary file 3 — Table S1. Description of symbols used. Table S2. Summary of descriptions and values corresponding to each trait. [file ece30004-0251-sd3.docx]

## Supplementary Tables

**Table S1.** Description of symbols used.

| **Symbol** | **Value(s)** | **Description** |
| --- | --- | --- |
| Traits | | |
| *A_pr_* | *α_pr_* | Probability of successfully acquiring prey item |
| *A_pl_* | *α_pl_* | Probability of successfully acquiring plant item |
| *B_pr_* | *β_pr_* | Proportion of total energy assimilated from prey resource |
| *B_pl_* | *β_pl_* | Proportion of total energy assimilated from plant resource |
| *G* | *γ* | Probability of engaging in an aggressive interaction |
| *R* | *ρ* | Allocation to reproduction (*i.e.*, offspring investment) |
| *D* | *δ* | Probability of moving from current location |
|  |  |  |
| Within-generation | | |
| *F(x)* | - | Fitness as a function of the size of an individual |
| *X* | *x* | Size of an individual |
| *N* | 1000 | Number of individuals in a simulation |
| *λ* | 0.50 | Proportion of loser's size lost to the winner in an aggressive interaction |
| *b_2_* | 5 | Slope of size allocation function |
| *b_1_* | 10 | y-intercept of size allocation function |
|  |  |  |
| Between-generation | | |
| *μ* | 0.02 | Probability of mutation |
| *ς* | 0.10 | Probability of recombination |
| *κ* | - | Difference function used in assortative mating routine |
| *S_i,j_* | - | Strategy *i*, (and trait *j*) used in the mating routine |
|  |  |  |
| Environmental gradients | | |
| *θ_i_* | 0.05 – 0.45 | Absolute resource availability (for resource *i*) |
| *φ_i_* | 20 – 100 | Absolute resource quality (for resource *i*) |
| *ω* | 1 – 40 | Relative resource availability |
| *ξ* | 10 | Relative resource quality |
|  |  |  |
| Size of world | | |
| *XMAX* × *YMAX* | 50×50 – 500×500 | Size of the environment in which simulations were conducted. |
|  |  |  |
| Costs | | |
| *ψ_i_* | - | Cost function for trait *i* |
| *c_A,pr_* | 0.01 | Proportional cost of acquiring prey resources |
| *c_A,pl_* | 0.01 | Proportional cost of acquiring plant resources |
| *c_B,pr_* | 0.01 | Proportional cost of assimilating prey resources |
| *c_B,pl_* | 0.01 | Proportional cost of assimilating plant resources |
| *c_G_* | 0.01 | Proportional cost of aggression |
| *c_X_* | 0.02 | Proportional cost of metabolism |
|  |  |  |
| Phenotype | | |
| *η* | - | Proportion of plants in the diet (i.e., phenotype) |
| *ϒ* | - | Feeding class (e.g., carnivore, omnivore, herbivore) |

**Table S2.** Summary of descriptions and values corresponding to each trait

| **Trait** | **Description** | **Trait Values** | | | | | | | |
| --- | --- | --- | --- | --- | --- | --- | --- | --- | --- |
|  |  | **0** | **1** | **2** | **3** | **4** | **5** | **6** | **7** |
| ***A_pr_*** | Probability of successfully acquiring prey item | 0.01 | 0.15 | 0.29 | 0.43 | 0.57 | 0.71 | 0.86 | 0.99 |
| ***A_pl_*** | Probability of successfully acquiring plant item | 0.01 | 0.15 | 0.29 | 0.43 | 0.57 | 0.71 | 0.86 | 0.99 |
| ***B_pr_*** | Proportion of total energy assimilated from prey resource | 0.01 | 0.15 | 0.29 | 0.43 | 0.57 | 0.71 | 0.86 | 0.99 |
| ***B_pl_*** | Proportion of total energy assimilated from plant resource | 0.01 | 0.15 | 0.29 | 0.43 | 0.57 | 0.71 | 0.86 | 0.99 |
| ***Z*** | Probability of engaging in an aggressive interaction | 0.01 | 0.33 | 0.66 | 0.99 | -- | -- | -- | -- |
| ***R*** | Allocation to reproduction (*i.e.*, offspring investment) | 10 | 15 | 20 | 25 | 30 | 35 | 40 | 45 |
| ***D*** | Probability of moving from current location | 0.125 | 0.250 | 0.375 | 0.500 | 0.625 | 0.750 | 0.875 | 1.000 |
